# Morphology- and size-dependent spectroscopic properties of Eu^3+^-doped Gd_2_O_3_ colloidal nanocrystals

**DOI:** 10.1007/s11051-014-2690-x

**Published:** 2014-10-18

**Authors:** Dominika Wawrzynczyk, Marcin Nyk, Artur Bednarkiewicz, Wiesław Strek, Marek Samoc

**Affiliations:** 1 Institute of Physical and Theoretical Chemistry, Wroclaw University of Technology, Wybrzeze Wyspianskiego 27, 50-370 Wroclaw, Poland; 2 Institute of Low Temperature and Structure Research, PAS, Okolna 2, 50-422 Wroclaw, Poland; 3 Wroclaw Research Centre EIT+, Stablowicka 147, 54-066 Wroclaw, Poland

**Keywords:** Oxide nanoparticles, Lanthanide luminescence, Judd–Ofelt theory, Z-scan technique, Nonlinear optics, Colloidal stability

## Abstract

The synthesis, morphological characterization, and optical properties of colloidal, Eu(III) doped Gd_2_O_3_ nanoparticles with different sizes and shapes are presented. Utilizing wet chemical techniques and various synthesis routes, we were able to obtain spherical, nanodisk, nanotripod, and nanotriangle-like morphology of Gd_2_O_3_:Eu^3+^ nanoparticles. Various concentrations of Eu^3+^ ions in the crystal matrix of the nanoparticles were tested in order to establish the levels at which the concentration quenching effect is negligible. Based on the luminescence spectra, luminescence lifetimes and optical parameters, which were calculated using the simplified Judd–Ofelt theory, correlations between the Gd_2_O_3_ nanoparticles morphology and Eu^3+^ ions luminescence were established, and allowed to predict the theoretical maximum quantum efficiency to reach from 61 to 98 %. We have also discussed the impact of the crystal structure of Gd_2_O_3_ nanoparticles, as well as coordinating environment of luminescent ions located at the surface, on the emission spectra. With the use of a tunable femtosecond laser system and the Z-scan measurement technique, the values of the effective two-photon absorption cross-section in the wavelength range from 550 to 1,200 nm were also calculated. The nonlinear optical measurements revealed maximum multi-photon absorption in the wavelength range from 600 to 750 nm.

## Introduction

The precise control of both morphology and spectroscopic properties of lanthanide-doped nanoparticles (NPs) is of great importance for their use in sensing and labeling applications. Among various factors pertinent to optimizing the properties, it is essential to consider the influence of NPs size and shape on spectroscopic properties of optically active ions embedded in the fabricated materials (Yan and Yan 2008; Zhang et al. 2009). With well-optimized synthesis protocols, it is possible to obtain NPs with narrow size and shape distributions, and further investigate their optical properties. Several reports showed the possibility of obtaining lanthanide-doped NPs with various morphologies such as spheres, plates, rods, nanotubes, nanowires, and even 3D flowers (Cao 2004; Gai et al. 2011; Paik et al. 2013; Paik and Murray 2013; Yang et al. 2008; Zheng et al. 2010). Additionally, the combination of unique optical features of lanthanide ions (Bünzli and Eliseeva 2011), i.e., long luminescence lifetimes, sharp excitation, and emission lines, with chosen crystal matrices and the nanometric size provides effective performance of the synthesized NPs. In particular, Eu^3+^ ions-doped oxide NPs have shown unique luminescent properties, owing to different possible narrowband luminescence excitation pathways. The Eu^3+^ ions have been excited by either direct 4f-4f electronic transitions in Eu^3+^ ions, through the host matrix absorption, Eu–O charge transfer, indirect Gd^3+^ or Ce^3+^ ions absorption, or even with the sensitization through the organic ligands attached to the NPs surface (Chen et al. 2013; Du et al. 2013; Grzyb et al. 2013; Kumar et al. 2013; Liu et al. 2006). As a result, the Eu^3+^ ions-doped oxide NPs showed intense, narrowband luminescence with excited state lifetimes up to 1 ms, suitable for applications as red emitting phosphors (Blasse 1994). Another interesting feature of Eu^3+^-doped oxide NPs is the possibility to excite the emission through multi-photon absorption processes. Although extensive literature regarding nonlinear optical (NLO) properties of Eu^3+^ organic complexes is available (Andraud and Maury 2009), the reports regarding NLO properties of inorganic lanthanide-doped NPs are scarce (Nyk et al. 2011; Wawrzynczyk et al. 2013b). The measured and calculated NLO parameters are usually limited to a single wavelength, thus the dispersions of these parameters are lacking, while wide-wavelength range measurements are essential for finding optimum wavelengths for multi-photon excitation of lanthanide luminescence. Additionally, since the 4f-4f photon transitions in lanthanides are two-photon allowed (Lakowicz et al. 2001; Sztucki and Strek 1986), the NLO spectroscopy can provide additional information regarding the electronic structure of the investigated materials.

Apart from being efficient luminescence centers, Eu^3+^ ions are also very good luminescent probes of local crystalline environment, which enable correlation between spectral and structural properties of lanthanide-doped hosts. It has been proved that the ratio of the intensities of ^5^ *D*_0_ → ^7^ *F*_2_ and ^5^ *D*_0_ → ^7^ *F*_1_ transitions in Eu^3+^ ions (asymmetry ratio, *R*) together with Judd–Ofelt intensity parameters (*Ω*_2_ and *Ω*_4_) can be considered indicative of the distortion in the symmetry of the local environment of the Eu^3+^ emitters (Boyer et al. 2004; Judd 1962; Kumar et al. 2013; Ofelt 1962). The intensity parameters *Ω*_2_ and *Ω*_4_ were successfully related with NaGdF_4_ and Gd_2_O_3_ NPs sizes (Bednarkiewicz et al. 2005; Liu et al. 2006), while Eu^3+^ ions luminescence was successfully used to probe the surface effects, originating from high surface to volume ratio in nanosized materials (Banski et al. 2013; Chang et al. 2007; Jia et al. 2010). Furthermore, Wiglusz et al. (Wiglusz et al. 2010) showed elegant correlation between Eu^3+^ luminescence decays and relative amount of Eu^3+^ ions located at different crystallographic sites of hydroxyapatites.

Here we report the synthesis and spectroscopic investigations of Gd_2_O_3_ NPs doped with Eu^3+^ ions. The obtained nanosized Gd_2_O_3_:Eu^3+^ NPs presented spherical, nanodisk, nanotripod, and nanotriangle-like morphologies. The various sizes and shapes of those NPs resulted from changes introduced in the thermal decomposition reaction, through modifying lanthanide precursors to capping ligands ratio, or by addition of Li^+^ ions. In order to perform detailed spectroscopic investigation, we have synthesized those NPs with various concentrations (0.5, 1, 2, 3, 4, and 5 mol%) of Eu^3+^ ions. The analysis of luminescence spectra, luminescence lifetimes, and Judd–Ofelt parameters revealed different concentration quenching for Eu^3+^ ions depending on the size and shape of Gd_2_O_3_ nanocrystals. We have also performed wide-wavelength range Z-scan measurements in order to calculate the values of effective two-photon absorption cross-section (*σ*_2,eff_) for representative Gd_2_O_3_ NPs of different shapes. The Z-scan experiments revealed multi-photon absorption in the wavelength range from 600 to 750 nm. Furthermore, the values of *σ*_2,eff_ and molecular mass scaled parameters (*σ*_2,eff_/M) obtained for Gd_2_O_3_:Eu^3+^ NPs were compared with the values obtained for other types of inorganic NPs.

## Experimental

### Materials and methods

Transmission Electron Microscopy (TEM) images and Selected Area Diffraction (SEAD) patterns were obtained by a FEI Tecnai G^2^ 20 X-TWIN microscope. X-ray powder diffraction (XRD) measurements were recorded on a STOE diffractometer with Ge-filtered CuK_α1_ radiation. The excitation spectra were recorded with a Hitachi F-4500 spectrofluorimeter. The emission spectra and luminescence decay curves were obtained under excitation from the third harmonic (266 nm) of a Ti:Sapphire laser pumped by a Nd:YAG LOTIS TII laser (Belarus). The luminescence signals were detected by a Synapse CCD camera (HORIBA JOBIN–YVON). Fluorescence decay curves were recorded with a photomultiplier (HAMAMATSU R928) whose output was fed into a LeCroy Wave Surfer 425 digital oscilloscope. The NLO measurements were performed using a femtosecond laser system consisting of a Quantronix Integra-C Ti:Sapphire regenerative amplifier which produces ~130 fs, 800 nm pulses with 1 kHz repetition rate, and 1 mJ energy per pulse, pumping a Quantronix Palitra-FS optical parametric amplifier (OPA). Details of the experiment were described in our previous papers (Nyk et al. 2011, 2012). Samples for Z-scan measurements were prepared in 1-mm-thick sealed glass cuvettes. Scans of the investigated Gd_2_O_3_:Eu^3+^ NPs were preceded by scans of a 4.6 mm-thick silica glass plate used as a reference, and a cuvette with pure solvent, the latter being used in order to eliminate the contributions of the cuvette walls and the solvent itself to the signals (Samoc et al. 2003). The open-aperture (OA) Z-scan traces were fitted using expressions derived by Sheik-Bahae (Sheikbahae et al. 1990). This allowed us to calculate the values of effective two-photon absorption cross-section *σ*_2,eff_ referring to individual Gd_2_O_3_:Eu^3+^ NPs.

### Synthesis of Eu^3+^ doped Gd_2_O_3_ nanocrystals

All chemicals for synthesis of Gd_2_O_3_:Eu^3+^ NPs were purchased from Sigma Aldrich or POCH S.A. (Poland), and used without further purification. In order to study the dependence of NPs sizes and shapes on Eu^3+^ optical properties, we synthesized Gd_2_O_3_ NPs with spherical, nanodisk, nanotripod, and nanotriangle-like NPs morphology. For nanosphere- and nanodisk-shaped Gd_2_O_3_ NPs, we utilized the synthesis protocol reported by Cao (Cao 2004). In a typical synthesis procedure, gadolinium acetate hydrate (1 or 2 mM) and europium acetate hydrate (1 or 2 mM) were dissolved in a mixture of 1.7 ml oleylamine, 1 ml oleic acid, and 2.7 ml octadecene at ~100 °C. The solution was degassed and heated to temperature around 300 °C, and then cooled to room temperature after 1 h. The obtained Gd_2_O_3_ NPs were precipitated with a mixture of methanol and acetone (2:1 v/v), and washed several times with methanol. Finally, the NPs were dissolved in 4 ml of CHCl_3_. For nanotripod- and nanotriangle-shaped Gd_2_O_3_ NPs, we followed the report by Paik et al. (Paik et al. 2013). In a typical synthesis procedure, lithium hydroxide (6 mM) was dissolved and degassed in a mixture of 12 ml of oleic acid, 18 ml of oleylamine, and 30 ml of 1-octadecene at ~100 °C. Next, gadolinium acetate (3 mM) and europium acetate (3 mM) were added into the solution, followed by the evaporation at ~100 °C. The yellowish, transparent solution was then heated to ~300 °C under N_2_ flow. Identical amounts of the reaction solution were picked up with a syringe after 45 min, 1.5, and 3.5 h, cooled to room temperature, precipitated with a mixture of methanol and acetone (2:1 v/v), and washed several times with methanol. Finally, the NPs were dissolved in 4 ml of CHCl_3_. The intentional Eu^3+^ ions content in the crystal matrix in the prepared samples was as follows: Gd_2_O_3_:x %Eu^3+^ (*x* = 0.5, 1, 2, 3, 4, and 5).

## Results and discussion

### Morphology and crystal structure

**Fig. 1:**
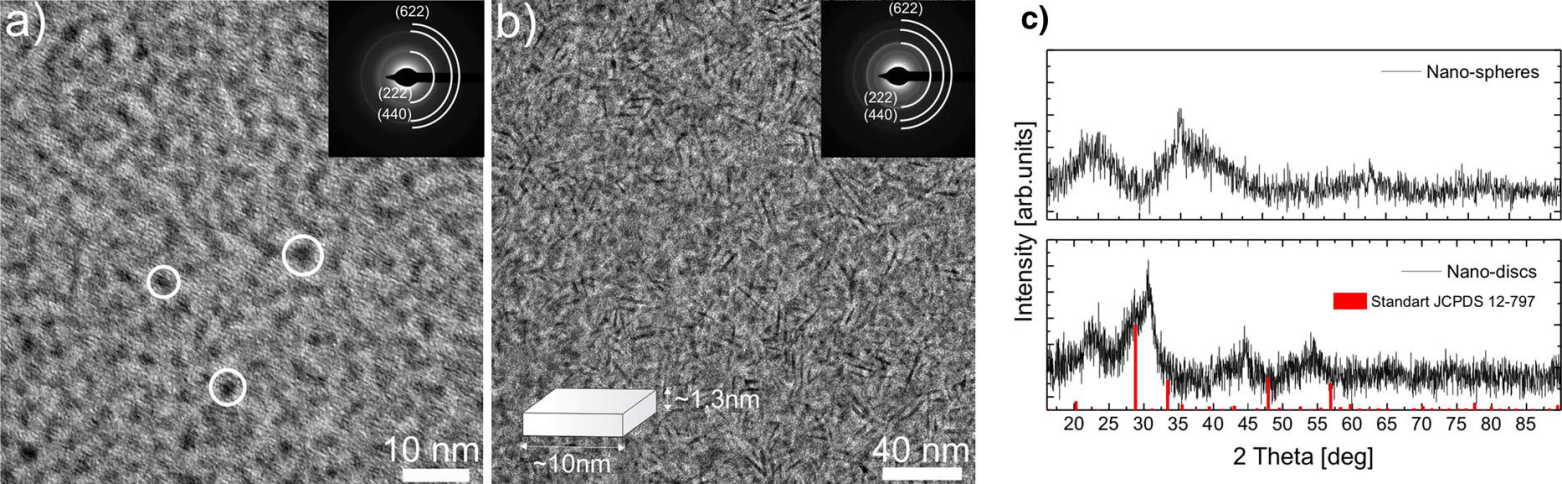
TEM images and SAED patterns for Gd_2_O_3_:Eu^3+^ NPs synthesized following (Cao 2004), with addition of 1 mM (**a**) and 2 mM (**b**) of lanthanide acetates. XRD patterns of Gd_2_O_3_:Eu^3+^ NPs with nanosphere and nano-disks like morphology compared with standard (JCPDS 12-797) pattern of cubic Gd_2_O_3_ (**c**)

**Fig. 2:**
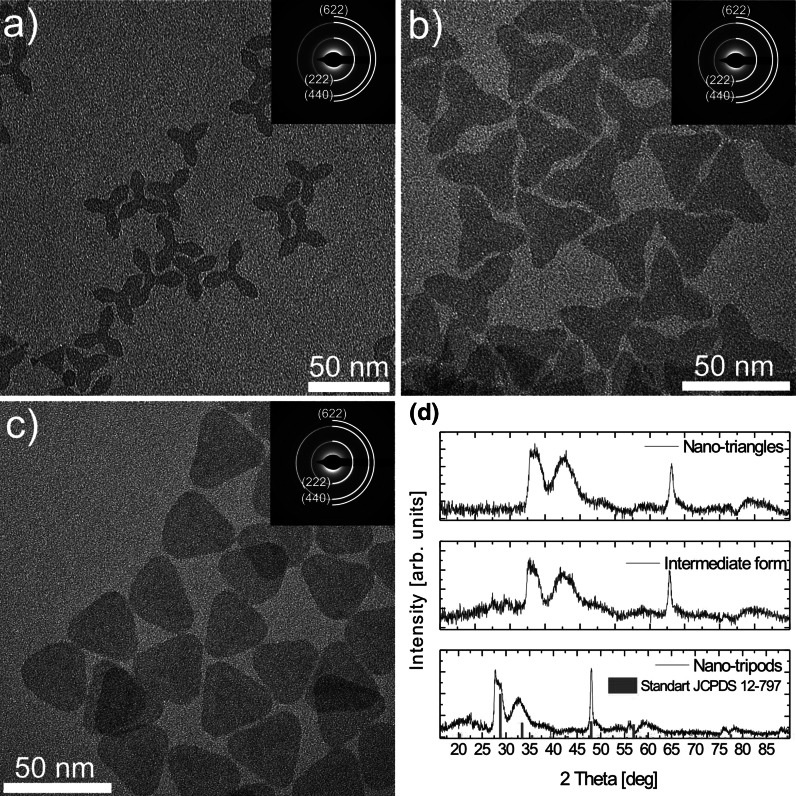
TEM images and SAED patterns for Gd_2_O_3_:Eu^3+^ NPs synthesized following Paik et al. (2013) obtained after 45 min (**a**), 1.5 h (**b**), and 3.5 h (**c**) time of synthesis. XRD patterns of nanotriangle, intermediate form, and nanotripod shaped Gd_2_O_3_:Eu^3+^ NPs compared with standard (JCPDS 12-797) pattern of cubic Gd_2_O_3_ (**d**)

Figures 1 and 2 present the morphological and crystallographic characterization of Eu^3+^-doped Gd_2_O_3_ NPs obtained by the synthesis protocols described by (Cao 2004) and (Paik et al. 2013), respectively. As can be seen from Fig. 1, when low volumes of coordinating solvents are used and no Li^+^ ions are added to the reaction, the synthesis results in formation of nanospheres and nanodiscs. In particular, smaller amount of Gd^3+^ and Eu^3+^ acetates (1 mM) with respect to the constant coordinating solvents volume results in formation of small, spherical Gd_2_O_3_ NPs with average sizes below 5 nm (Fig. 1a). The increase of the lanthanide acetates amount (2 mM) results in formation of thin nanodiscs with average dimensions ~10 nm/~1.3 nm (Fig. 1b). Based on the SAED patterns (insets in Fig. 1a, b), and comparisons of XRD patterns for nanospheres and nanodiscs (Fig. 1c) with standard (JCPDS 12-797) pattern of cubic Gd_2_O_3_, we have assigned the obtained NPs to the Gd_2_O_3_ cubic phase. We have observed significant broadening of the XRD peaks and diffraction rings diameters related to the lattice plane spacing, being a result of small sizes of the NPs.

**Fig. 3:**
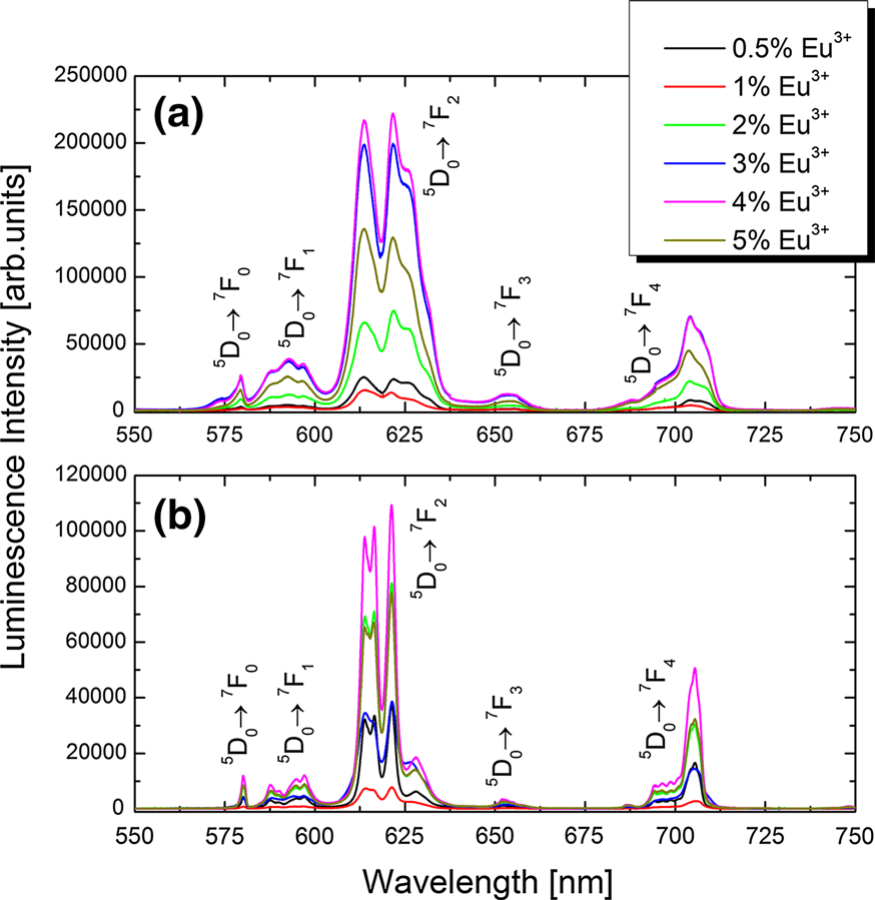
Luminescence spectra of Gd_2_O_3_:Eu^3+^ nanospheres (**a**) and nanodiscs (**b**) doped with different amounts of Eu^3+^ ions; measured at room temperature under 266 nm laser light excitation

Introducing Li^+^ ions into the reaction results in the formation of thin nanotripod- and nanotriangle-shaped Gd_2_O_3_:Eu^3+^ NPs. As it was previously shown by Paik et al. (2013), by prolonging the reaction time the NPs shape is transformed from nanotripods (Fig. 2a) to nanotriangles (Fig. 2c) throughout the transitional form (Fig. 2b). The obtained Gd_2_O_3_:Eu^3+^ NPs possessed much narrower size and shape distribution and, in comparison with previously obtained nanospheres and nanodiscs, were much bigger. The average diameter of Gd_2_O_3_:Eu^3+^ NPs was ~40 nm. Based on the SAED (insets in Fig. 2a–c) and XRD patterns (Fig. 2d), we could again assign the obtained NPs to the Gd_2_O_3_ cubic form, as expected for the case of bigger NPs, and the diffraction pattern rings were sharp and more intense. The broadening of the peaks at the XRD patterns results from the small thickness of obtained Gd_2_O_3_:Eu^3+^ NP, which did not exceed 2 nm. The influence of the Li^+^ ions on the size and shape of the obtained Gd_2_O_3_ NPs may be related to the direct doping of Li^+^ ions into the crystal matrix, causing lattice expansion (Paik et al. 2013). Extensive work concerning the influence of alkali ions (Li^+^, Na^+^, and K^+^) on the formation of fluoride NPs has been performed by (Xue et al. 2013) showing that, depending on the ionic radius of the added alkali ions, formation of various fluoride NPs morphologies may occur.

### Photoluminescence properties and Judd–Ofelt parameters of Gd_2_O_3_:Eu^3+^ nanocrystals

**Fig. 4:**
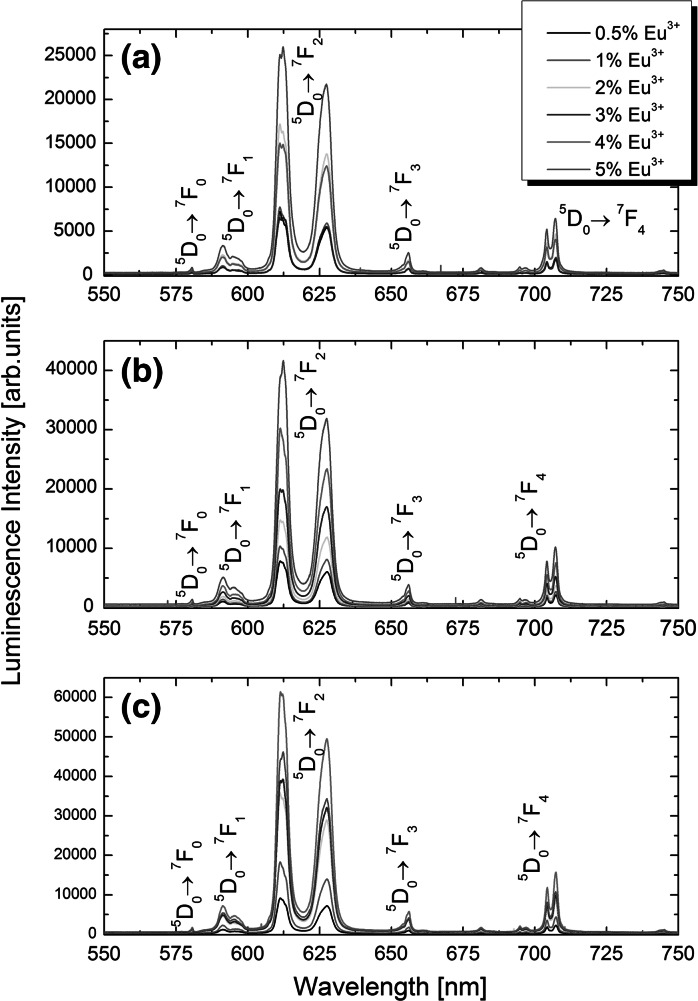
Luminescence spectra of nanotripods (**a**) intermediate form (**b**) and nanotriangles (**c**) shaped Gd_2_O_3_ NPs doped with different amounts of Eu^3+^ ions and excited with 266 nm laser light

**Fig. 5:**
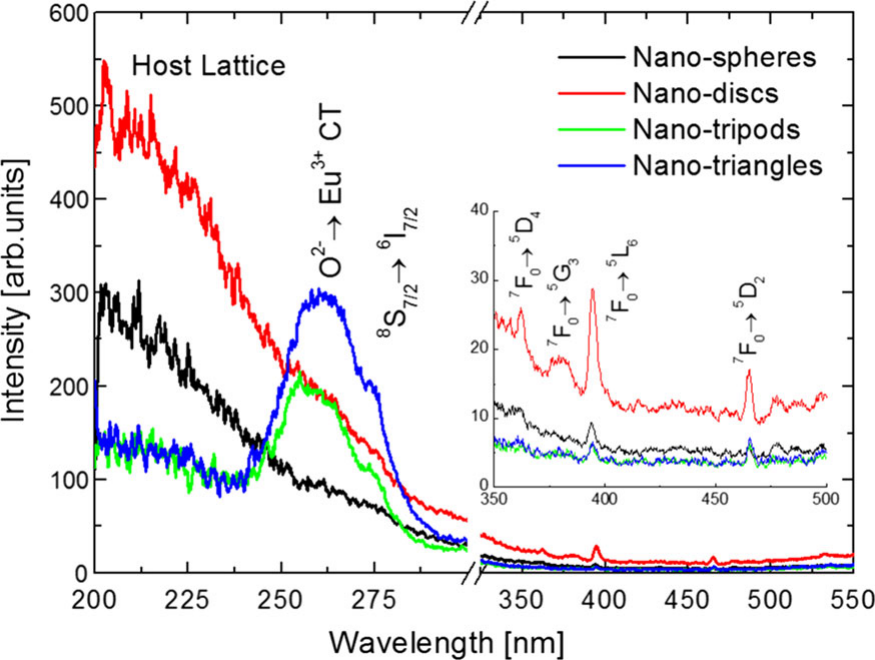
Excitation spectra of representative Gd_2_O_3_:Eu^3+^ NPs with the shapes of nanospheres, nanodiscs, nanotripods, and nanotriangles obtained by monitoring ^5^ *D*_0_ → ^7^ *F*_2_ transition of Eu^3+^ emission at 613 nm

The main purpose of this work was to search for correlation between spectroscopic properties of Eu^3+^ ions doped the Gd_2_O_3_ matrix with the size and shape of the respective NPs. The investigated Gd_2_O_3_:Eu^3+^ NPs showed intense red luminescence under 266 nm laser excitation, and the observed luminescence lines (Figs. 3, 4) resulted from the electronic 4f-4f transitions from ^5^ *D*_0_ level of Eu^3+^ ions to ^7^ *F*_J=0,1,2,3,4_ ground state components, from which the most intensive ones could be observed at ~613 and ~625 nm (^5^ *D*_0_ → ^7^ *F*_2_). In order to study the possible Eu^3+^ luminescence excitation pathways, we recorded excitation spectra, monitoring the most intense emission line at 613 nm (^5^ *D*_0_ → ^7^ *F*_2_). The representative results for nanospheres, nanodiscs, nanotripods, and nanotriangles are shown in Fig. 4. The excitation spectra are dominated by the Gd_2_O_3_ (maximum at ~200 nm) host absorption for smaller nanospheres and nanodiscs, and by the charge-transfer (CT) absorption (~260 nm) for the nanotripods and nanotriangles with larger average sizes. With increasing the size of Gd_2_O_3_:Eu^3+^ NPs, the excitation from host matrix gets less dominant, and the CT band occurs. This fact can be well understood, since the CT relies on electron transfer between the O^2-^ and Eu^3+^, and many more of such optically active centers can be found in bigger NPs. A similar effect was observed by (Li and Hong 2007) for nanocrystalline Gd_2_O_3_:Eu^3+^ powders, where the ratio of host absorption to CT band of smaller NPs was much bigger, compared with that for larger NPs. Additionally, we could observe characteristic narrowband excitation peaks corresponding to the direct 4f-4f electronic transitions in Gd^3+^ (^8^ *S*_7/2_ → ^6^ *I*_7/2_) and in the Eu^3+^ ions (inset in the Fig. 5). The intensity of the excitation peak from the Gd^3+^ ions was much higher than for the case of Eu^3+^ ions, owing to very low (from 0.5 to 5 %) Eu^3+^ ions concentration in the Gd_2_O_3_ matrix.

**Fig. 6:**
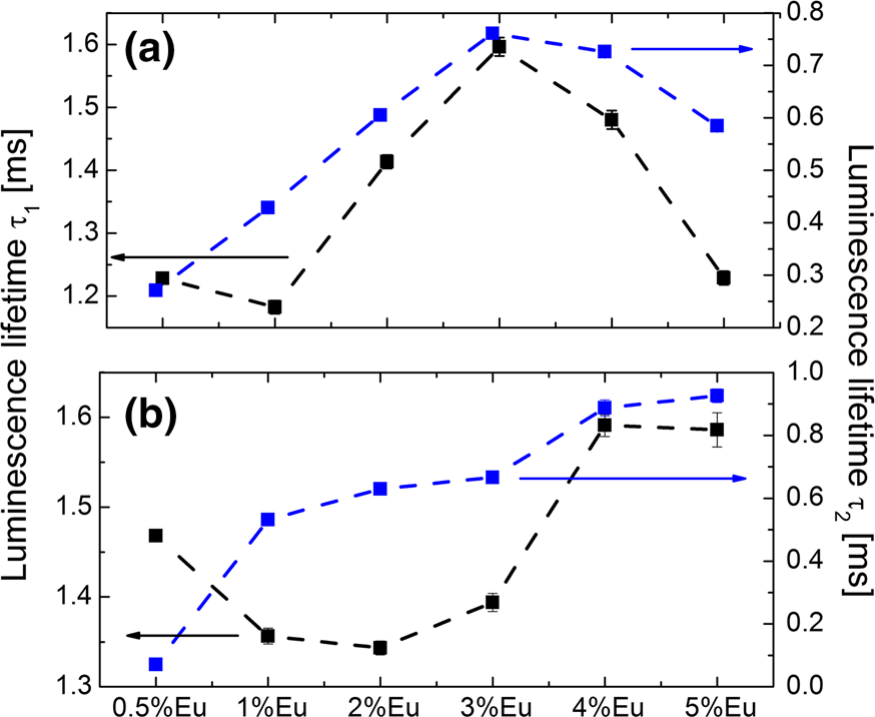
Luminescence lifetime values corresponding to the long *τ*_1_ (*black*) and short *τ*_2_ (*blue*) components for ^5^ *D*_0_ excited state in Eu^3+^ ions measured for Gd_2_O_3_:Eu^3+^ nanospheres (**a**) and nanodiscs (**b**) excited with 266 nm laser light. (Color figure online)

In order to find Eu^3+^ ions doping level at which the concentration quenching is the weakest in the Gd_2_O_3_ matrix, as well as to investigate the influence of nanocrystals’ shape and crystal structure on Eu^3+^ emission, we have measured luminescence spectra and ^5^ *D*_0_ excited state lifetimes for Gd_2_O_3_:Eu^3+^ NPs of different shapes, as a function of rising Eu^3+^ concentrations. In the case of nanospheres, the observed luminescence lines were considerably broadened in comparison with the larger Gd_2_O_3_:Eu^3+^ NPs (Fig. 3a). Those Gd_2_O_3_:Eu^3+^ NPs had the highest surface to volume ratio, and the largest number of Eu^3+^ ions was exposed to the interaction with organic ligands as well as solvent molecules (Liu et al. 2006; Wawrzynczyk et al. 2013a). The slight increase in Gd_2_O_3_:Eu^3+^ NPs’ size resulted in evident narrowing of the emission lines for the nanodiscs, nanotripods, and nanotriangles (Fig. 3b and Fig. 4). We have also observed roughly even intensity of the two peaks characteristic for the ^5^ *D*_0_ → ^7^ *F*_2_ electronic transition in Eu^3+^ ions. The Gd_2_O_3_ NPs are obtained either in the monoclinic or cubic crystal structure of nanopowders, with emission spectra maximum at longer wavelengths (~620 nm) for the monoclinic form, and blue-shifted (~610 nm) for the cubic one (Seo et al. 2009). The increased ratio of Eu^3+^ emission intensities at 620 nm to the peak at 610 nm for the investigated cubic Gd_2_O_3_ NPs may be rationalized by high surface to volume ratio. In such a case, a significant number of Eu^3+^ ions are located at NPs’ surface, and thus, Eu^3+^ display a lower coordination number and emission spectra similar to monoclinic polycrystalline powders, in which Eu^3+^ ions occupy lower symmetry. The same relationships were observed by (Si et al. 2005) for Eu_2_O_3_ NPs. Gd_2_O_3_:Eu^3+^ colloidal NPs emission spectra, with peaks at 610 and 620 nm having roughly even intensities, have been also reported by (Paik et al. 2013). The highest luminescence intensity was observed at 4 % Eu^3+^ doping level for nanospheres and nanodiscs (Fig. 3), at 5 % for nanotripods and at 4 % for nanotriangles (Fig. 4). However, these values were found somewhat susceptible to the concentration of the Gd_2_O_3_:Eu^3+^ NPs in the chloroform colloidal dispersion, sample experimental setup alignment, and fluctuation in the excitation light intensity.

The luminescence lifetime measurements provide more reliable information regarding optimum Eu^3+^ concentration in the host matrix. In general, longer ^5^D_0_ excited state luminescence lifetimes indicate lower quenching arising from surface defects, vibrational modes of ligands molecules, and impurities inside the crystal matrix. Apart from these external quenching mechanisms, additional concentration quenching may occur in heavily Eu^3+^ doped hosts, which originates from the distance dependent non-radiative cross relaxation between neighbor Eu^3+^ ions (Blasse 1994; Singh et al. 2008). For nanospheres and nanodiscs, the observed luminescence decays were double exponential, with the short lifetime component in the range from 271 ± 4 μs up to 761 ± 5 μs, and from 535 ± 6 μs up to 926 ± 2 μs in the case of nanospheres and nanodiscs, respectively (Fig. 6). The long luminescence lifetime components varied in the range from 1.18 ± 0.01 μs up to 1.60 ± 0.02 μs, and from 1.34 ± 0.01 μs up to 1.59 ± 0.02 μs in the case of nanospheres and nanodiscs, respectively (Fig. 6). The longest decay times, for both *τ*_1_ and *τ*_2_, were measured for 3 and 4 % of Eu^3+^ doping level concentration for nanospheres and nanodiscs, respectively. The relative maximum changes of the measured long lifetime components in relation to the Eu^3+^ level concentration in the matrix were found to be ~30 and ~20 % for nanospheres and nanodiscs, respectively.

**Fig. 7:**
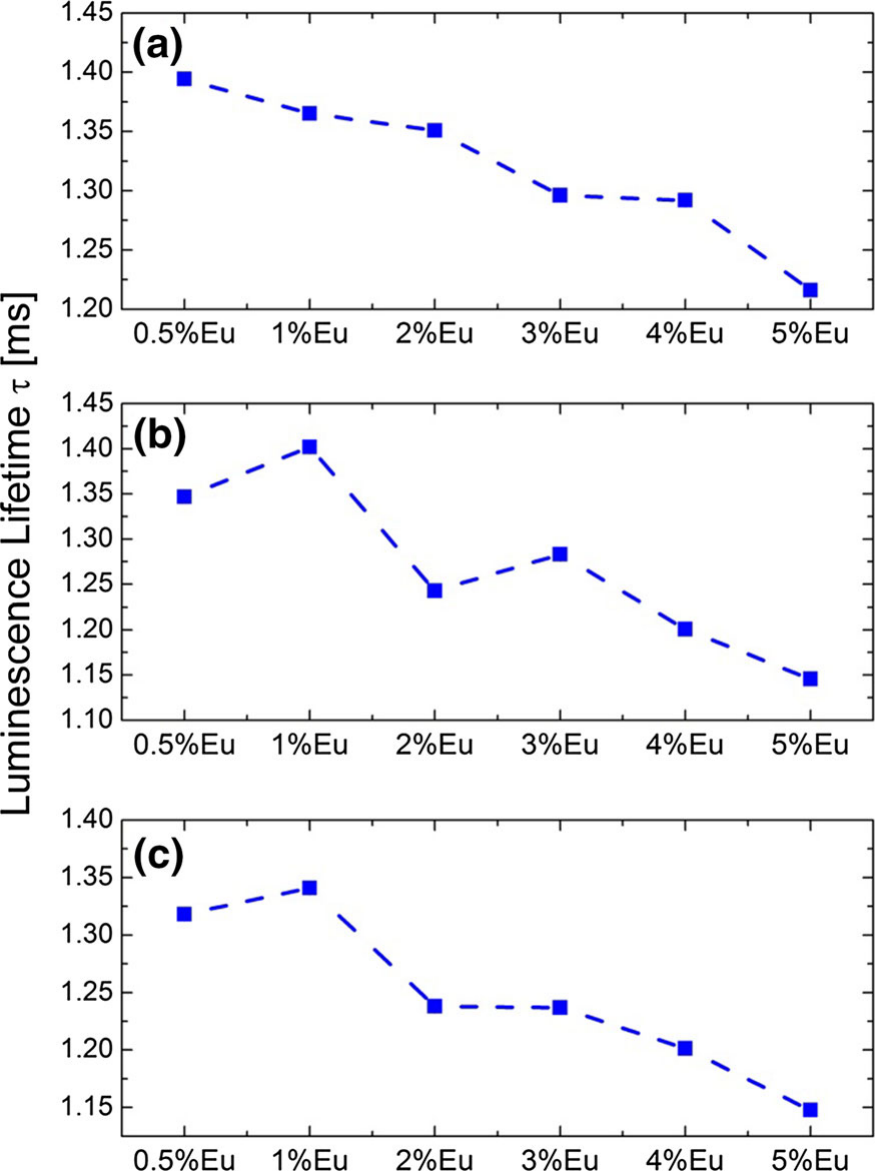
Luminescence lifetimes values of ^5^D_0_ excited state in Eu^3+^ ions measured for Gd_2_O_3_ NPs shaped as nanotripods (**a**), intermediate form (**b**), and nanotriangles (**c**) with 266 nm laser light excitation

For bigger Gd_2_O_3_:Eu^3+^ NPs shaped as nanotripods and nanotriangles, the observed luminescence decays were single exponential (Fig. 7), and the lifetimes varied in the range from 1.22 ± 0.01 μs up to 1.39 ± 0.01 μs, from 1.15 ± 0.01 μs up to 1.40 ± 0.01 μs, and from 1.15 ± 0.01 μs up to 1.34 ± 0.01 μs, for nanotripods, intermediate form (nanotripods → nanotriangles), and nanotriangles, respectively. The longest luminescence lifetime values were measured for 0.5 %Eu^3+^ doping for nanotripods, and for 1 %Eu^3+^ for nanotriangles, and intermediate form of Gd_2_O_3_ NPs. The relative maximum rate of changes in measured luminescence lifetimes varied in the range from ~10 to 20 %, with changing the Eu^3+^ doping level in the Gd_2_O_3_ crystal matrix. The less significant changes of the observed Eu^3+^ ^5^ *D*_0_ excited state lifetimes in bigger Gd_2_O_3_ NPs show that in NPs with lower surface to volume ratio (i.e., in larger NPs) the Eu^3+^ ions are less susceptible to the changes in the local environment.

The observed lower Eu^3+^ concentration quenching in nanotripods and nanotriangles could be attributed to the size of the Gd_2_O_3_ NPs. In smaller nanosized particles, the non-radiative quenching centers distribute randomly with a considerable fluctuation within the particle, and at the same time, the increased surface quenching effect in smaller particles effectively competes with cross-relaxation between Eu^3+^ ions (Li and Hong 2007). Additionally, in smaller NPs, the quenching traps distribute randomly, and resonance energy transfer between neighbor Eu^3+^ ions can occur only within one particle due to the hindrance by the particle surface. When increasing the concentration of luminescence centers, quenching occurs first in particles containing many traps, while those particles containing fewer traps quench only at high concentration (Jiang et al. 2003). Therefore, quenching occurs preferentially at higher Eu^3+^ concentration in smaller NPs. For gaining better insight into the spectroscopic properties of synthesized Gd_2_O_3_:Eu^3+^ NPs, we have also calculated and analyzed Judd–Ofelt intensity parameters (Judd 1962; Ofelt 1962).

The Judd–Ofelt intensity parameters: *Ω*_2_ and *Ω*_4_, can be used to investigate the spectroscopic properties of Eu^3+^ ions embedded in Gd_2_O_3_ crystal matrix. Following Kodaira et al. (Kodaira et al. 2003), we have calculated the *Ω*_2_ and *Ω*_4_ parameters, as well as transition rates and quantum efficiencies, from emission spectra and luminescence decays time. The proposed approach uses the integral intensity of ^5^ *D*_0_ → ^7^ *F*_1_ magnetic dipole transition as a reference for calculating experimental intensity parameters for ^5^ *D*_0_ → ^7^ *F*_2_ and ^5^ *D*_0_ → ^7^ *F*_4_ ones. According to the Judd–Ofelt theory, the rates of ^5^ *D*_0_ → ^7^ *F*_J_ transition can be expressed as

1 $$ A_{0J} = \frac{{64\pi^{4} \upsilon_{J}^{3} }}{{3h\left( {2J + 1} \right)}}e^{2} \frac{{n\left( {n^{2} + 2} \right)^{2} }}{9}\mathop {\sum_{\lambda = 2,4,6} }\limits \,\varOmega_{\lambda } \left| {\langle \,^{5} D_{0} ||U^{\left( \lambda \right)} ||\,^{7} F_{J} \rangle } \right|^{2} , $$

where *e* is the electronic charge, *ν*_*J*_ is the wavenumber of the corresponding transition, (2 J + 1) is equal to 1 for ^5^ *D*_0_ transition, *h* is the Planck constant, and *n* is the refractive index of the investigated material. For calculations of the effective refractive index (Meltzer et al. 1999) of the investigated CHCl_3_ solution of Gd_2_O_3_:Eu^3+^ NPs, we have estimated the filling factor to be approximately 5 %, based on Gd_2_O_3_:Eu^3+^ NPs morphology measurements and gravimetric analysis after evaporating solvent from a certain amount of the solution. The wavelength dependent refractive index of cubic Gd_2_O_3_ was calculated using Sellmeier equation (Liu et al. 2007; Medenbach et al. 2001). 〈^5^ *D*_0_||*U* ^(*λ*)^||^7^ *F*_*J*=2,4_〉 are the squared reduced matrix elements, with values independent of the chemical environment of the Eu^3+^ ions, and equal to 0.0032 and 0.0023 for *J* = 2 and 4 (Kaminskii 1996), respectively. The *A*_0–1_ coefficient is a constant and independent of the medium, being equal to 50 s^−1^ (Hreniak et al. 2004). The relation between the integral intensity of transition (*I*_0-J_), the transition energy (*h*υ_0–J_), emission coefficient (*A*_0–J_), and ^5^ *D*_0_ energy level population *N* is *I*_0–J_ = *h*υ_0–J_ *A*_0–J_ *N*. Comparison of the relation for ^5^ *D*_0_ → ^7^ *F*_2_ and ^5^ *D*_0_ → ^7^ *F*_4_ transitions versus ^5^ *D*_0_ → ^7^ *F*_2_ allows one to rewrite the expression for *A*_0–J_ as

2 $$ A_{0 - J} = A_{0 - 1} \frac{{I_{0 - J} }}{{I_{0 - 1} }}\frac{{\upsilon_{0 - J} }}{{\upsilon_{0 - 1} }}. $$

Combination of Eq. (1) and (2) allows for calculation of *Ω*_2_ and *Ω*_4_ intensity parameters, which are collected in Table 1. Table 1 presents also emission quantum efficiency (*η*), lifetimes (*τ*), non-radiative (*A*_nrad_), radiative (*A*_rad_), and total (*A*_total_) decay rates for the observed transitions in all the investigated Eu^3+^ doped Gd_2_O_3_ NPs of different shapes. The above spectroscopic parameters have been calculated based on following equations: $A_{rad} = \sum _{j} A_{0 - J}$, $A_{total} = \frac{1}{\tau } = A_{rad} + A_{nrad}$, $\eta = \frac{{A_{rad} }}{{A_{rad} + A_{nrad} }}.$ In the case of nanospheres and nanodiscs, which showed double-exponential luminescence decays, we have calculated the average luminescence lifetime (*τ*_avg_) based on equation: *τ*_avg_ = (*A*_1_ *τ*_1_ + *A*_2_ *τ*_2_)/(*τ*_1_ + *τ*_2_), where A_1_ and A_2_ are the amplitudes of long and short decay components, respectively.

**Table 1:** Judd–Ofelt analysis of Eu^3+^ doped Gd_2_O_3_ NPs with different shapes: intensity parameters (*Ω*_2_ and *Ω*_4_), emission quantum efficiency (*η*), lifetimes (*τ*), non-radiative (*A*_nrad_), radiative (*A*_rad_), and total (*A*_total_) rates for observed transitions

Samples Gd_2_O_3_:x %Eu^3+^	*Ω*_2_ (10^−20^ cm^2^)	*Ω*_4_ (10^−20^ cm^2^)	*A*_rad_ (s^−1^)	*A*_nrad_ (s^−1^)	*A*_total_ (s^−1^)	*τ* (ms)	*η* (%)
Nanospheres							
0.5 % Eu^3+^	11.7	5.10	502.4	688.1	1190.5	0.84	42.2
1 % Eu^3+^	9.3	4.58	419.9	1095.2	1515.0	0.66	27.7
2 % Eu^3+^	12.8	4.92	520.2	714.4	1234.6	0.81	42.1
3 % Eu^3+^	12.1	5.26	505.4	484.7	990.1	1.01	51.1
4 % Eu^3+^	12.8	5.08	520.5	521.2	1041.8	0.96	50.0
5 % Eu^3+^	11.3	5.39	496.2	738.3	1234.6	0.81	40.2
Nanodiscs							
0.5 % Eu^3+^	13.4	7.65	583.8	1029.0	1612.8	0.62	36.2
1 % Eu^3+^	15.0	7.10	623.9	566.6	1190.5	0.84	52.4
2 % Eu^3+^	14.7	7.52	622.8	286.3	909.1	1.10	68.5
3 % Eu^3+^	13.8	6.55	577.0	443.4	1020.4	0.98	56.6
4 % Eu^3+^	14.5	8.52	633.8	80.5	714.3	1.40	88.7
5 % Eu^3+^	13.3	7.60	580.6	182.8	763.4	1.31	76.1
Nanotripods							
0.5 % Eu^3+^	14.6	5.41	603.0	116.4	719.4	1.39	83.8
1 % Eu^3+^	15.4	5.57	629.7	100.2	729.9	1.37	86.3
2 % Eu^3+^	16.3	5.64	661.6	79.2	740.7	1.35	89.3
3 % Eu^3+^	16.1	5.52	582.3	186.9	769.2	1.30	75.7
4 % Eu^3+^	16.3	5.50	586.8	188.4	775.2	1.29	75.7
5 % Eu^3+^	17.1	5.49	683.1	136.5	819.7	1.22	83.3
Intermediate (Nanotripods → Nanotriangles)							
0.5 % Eu^3+^	15.1	5.66	623.5	117.3	740.7	1.35	84.2
1 % Eu^3+^	16.4	5.38	658.1	56.2	714.3	1.40	92.1
2 % Eu^3+^	16.8	5.70	676.6	129.9	806.5	1.24	83.9
3 % Eu^3+^	16.7	5.72	672.8	108.5	781.3	1.28	86.1
4 % Eu^3+^	17.5	5.46	695.7	137.6	833.3	1.20	83.5
5 % Eu^3+^	16.3	5.62	660.8	208.8	869.6	1.15	76.0
Nanotriangles							
0.5 % Eu^3+^	14.9	5.57	615.3	142.3	757.6	1.32	81.2
1 % Eu^3+^	16.7	5.49	668.8	77.5	746.3	1.34	89.6
2 % Eu^3+^	15.9	5.53	645.7	160.7	806.5	1.24	80.1
3 % Eu^3+^	16.6	5.70	670.4	136.1	806.5	1.24	83.1
4 % Eu^3+^	18.2	5.64	720.9	112.4	833.3	1.20	86.5
5 % Eu^3+^	16.6	5.47	666.5	203.1	869.6	1.15	76.7

The *Ω*_2_ intensity parameter reflects the hypersensitive behavior of the ^5^ *D*_0_ → ^7^ *F*_2_ transitions, and the observed relatively high *Ω*_2_ values, when compared to Eu^3+^ doped NaYF_4_ NPs (Bednarkiewicz et al. 2005) result from the presence of highly electronegative O^2-^ ions in the Gd_2_O_3_ crystal structure. We have also observed slightly lower *Ω*_2_ values for nanosphere and nanodisc-like shaped Gd_2_O_3_:Eu^3+^ NPs in comparison with Gd_2_O_3_:Eu^3+^ NPs of other shapes. It may be due to the fact, that in smaller NPs, the lanthanide ion tends to increase its local symmetry (Malyukin et al. 2003). Additionally, in the synthesis of nanotripods, intermediate form, and nanotriangles, Li^+^ ions have been used, and since the ionic radius of this alkali metal is different from Gd^3+^ ions, the crystal lattice is distorted, and therefore, the symmetry around the Eu^3+^ ions can be disturbed. The observed higher *Ω*_2_ values for bigger Gd_2_O_3_:Eu^3+^ NPs can be thus indicative of efficient incorporation of Li^+^ ions inside the crystal structure of matrix. Additionally, the colloidal character of studied Gd_2_O_3_:Eu^3+^ NPs and influence of organic ligands attached to the NPs surface can influence the values of *Ω*_2_. Kumar et al. (Kumar et al. 2013) reported lower *Ω*_2_ values for nanosized Gd_2_O_3_:Eu^3+^ NPs; however, those nanophosphors did not have any organic molecules attached to the surface. In the case of *Ω*_4_ intensity parameter, no size or shape dependency was observed. Only for nanodiscs the values of *Ω*_4_ were slightly higher.

It is also very important to study the influence of both Gd_2_O_3_:Eu^3+^ NPs size and shape on the radiative contribution for the depopulation of the emitting level, and consequently for the emission quantum efficiency. *A*_rad_ was higher for nanotripods and nanotriangles, when compared to the nanospheres, with simultaneous decrease of non-radiative contribution. This is why the relatively high η values, ranging from ~60 % for nanospheres up to ~90 % for nanotripods, were observed suggesting high efficiencies of energy transfer and efficient Eu^3+^ luminescence excitation in Gd_2_O_3_ matrix of those shapes.

### Nonlinear optical properties of Gd_2_O_3_:Eu^3+^ nanocrystals

**Fig. 8:**
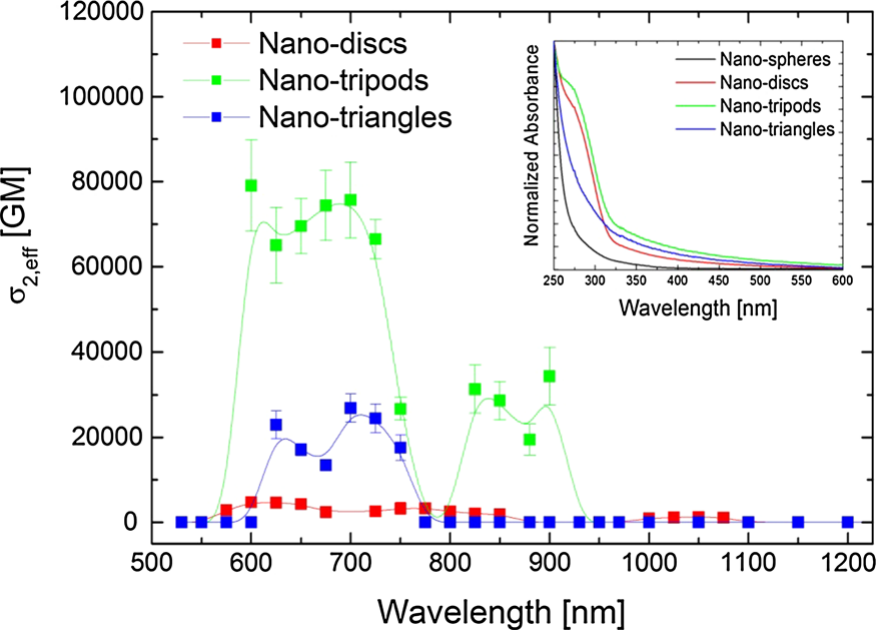
The wavelength dependence of the effective two-photon absorption cross-section *σ*_2,eff_ for Gd_2_O_3_:Eu^3+^ NPs shaped as nanodiscs (*red*), nanotripods (*green*), and nanotriangles (*blue*). The *inset* shows linear absorption spectra of the investigated Gd_2_O_3_:Eu^3+^ NPs. (Color figure online)

For complex spectroscopic characterization of the synthesized Gd_2_O_3_:Eu^3+^ NPs, we have also performed measurements of wavelength dispersion of the effective two-photon absorption cross-section (*σ*_2,eff_). Based on our previous results (Wawrzynczyk et al. 2013b), which showed that NLO absorption in YVO_4_ NPs is mainly dominated by NLO absorption through the CT transitions, and the contribution from 4f-4f transitions in Eu^3+^ ions is negligible, we have selected four representative Gd_2_O_3_:Eu^3+^ NPs shaped as nanospheres, nanodiscs, nanotripods, and nanotriangles for Z-scan measurements. Concentration of the selected samples was estimated through gravimetric analysis after evaporating the solvent from a known amount of the solution. Concentrations of the samples were ~67, 89, 25, and 45 mg/ml for nanospheres, nanodiscs, nanotripods, and nanotriangles, respectively. Density of bulk cubic Gd_2_O_3_ and mean sizes estimated based on TEM images of individual Gd_2_O_3_:Eu^3+^ NPs were used to calculate the molar mass (M) of a single Gd_2_O_3_:Eu^3+^ NP. The determined values allowed us to calculate the NLO parameters, and the appropriate merit factors for quantitative comparisons between different types of lanthanide-doped NPs, quantum dots (QDs), and plasmonic NPs. The results of wide-wavelength studies of NLO absorption of the selected Gd_2_O_3_:Eu^3+^ NPs are plotted in Fig. 8, as the effective two-photon absorption cross sections taken per single Gd_2_O_3_:Eu^3+^ NP. The smallest nanosphere-shaped Gd_2_O_3_:Eu^3+^ NPs did not show measurable NLO absorption in the whole spectral range. In general, the strength of the NLO absorption, presented as the *σ*_2,eff_ value, scales with the NPs size, and since those Gd_2_O_3_:Eu^3+^ NPs had mean sizes smaller than 5 nm, a weak excitation band in comparison with larger investigated NPs (Fig. 4) together with linear absorption spectra dominated by scattering (inset in Fig. 8) could result in lack of observable NLO absorption in nanosphere-shaped Gd_2_O_3_:Eu^3+^ NPs.

The nanodisc-, nanotripod-, and nanotriangle-shaped Gd_2_O_3_:Eu^3+^ NPs showed well-defined NLO absorption in the wavelength range between 600 nm and 750 nm (Fig. 8). Additionally, the nanotripod-shaped Gd_2_O_3_:Eu^3+^ NPs exhibited a second band in the *σ*_2,eff_ vs. λ plot in the range between 800 nm and 950 nm. The actual mechanisms responsible for the non-linear absorption occurring within the observed NLO absorption bands are likely to be quite complicated, possibly involving both two- and three-photon transitions, thus one should treat the values given here as effective ones, referring to intensities of about 100 GW/cm^2^, with possible more complex intensity dependences. The transitions are likely to have components due to both the host matrix absorption and the CT bands. In order to quantitatively compare the NLO properties of studied Gd_2_O_3_:Eu^3+^ NPs with different inorganic nanostructures, we have collected the calculated peak values of *σ*_2,eff_ and *σ*_2,eff_/M in Table 2.

**Table 2:** Comparison of NLO parameter *σ*_2,eff_ and merit factor *σ*_2,eff_/M for different types of inorganic nonlinear absorbers

Nanostructure	Wavelength (nm)	*σ*_2,eff_ (GM)	*σ*_2,eff_/M (GM mol/g)	Reference
Nano-disk-shaped Gd_2_O_3_:Eu^3+^ NPs	600	4,700	0.01	This work
Nanotripod-shaped Gd_2_O_3_:Eu^3+^ NPs	700	7,600	0.03	This work
Nanotriangle-shaped Gd_2_O_3_:Eu^3+^ NPs	700	26,700	0.01	This work
NaYF_4_:2 %Er^3+^, 20 %Yb^3+^ NPs	980	8,000,000	0.1	Nyk et al. (2011)
YVO4:Eu^3+^ NPs	650	720,000	0.09	Wawrzynczyk et al. (2013b)
CdSe quantum dots (4.2 nm)	970	66,000	0.13	Nyk et al. (2012)
CdS quantum dots (5 nm)	750	7,200	0.04	Szeremeta et al. (2013)
10 × 35 nm gold nanorods	850	21,000,000	14	Olesiak-Banska et al. (2012)

For the case of the studied in this work Gd_2_O_3_:Eu^3+^ NPs of different shapes the most significant values of *σ*_2,eff_ were ~4,700, 7,600, ~26,700 GM for nanodiscs, nanotripods, and nanotriangles at 600, 700, and 700 nm, respectively. For the sake of the comparisons of multiple photon absorption merit, one needs to scale the values of *σ*_2,eff_ with the Gd_2_O_3_:Eu^3+^ NPs size. The calculated merit factors *σ*_2,eff_/M (M being the molecular weight of a particle) were ~0.01, 0.03, 0.01 for nanodiscs, nanotripods, and nanotriangles, respectively. These values are therefore an order of magnitude lower than the corresponding ones for YVO_4_ (Wawrzynczyk et al. 2013b) and NaYF_4_ (Nyk et al. 2011) lanthanide-doped NPs, and two orders of magnitude lower than those for some two-photon absorbing chromophores (Schwich et al. 2011). However, the 5 nm large CdS quantum dots exhibited similar value of *σ*_2,eff_/M merit factor. In comparison with lanthanide-doped NPs, previously investigated in our group (Nyk et al. 2011; Wawrzynczyk et al. 2013b), as well as quantum dots and metallic NPs (Nyk et al. 2012; Olesiak-Banska et al. 2012; Szeremeta et al. 2013), the Gd_2_O_3_:Eu^3+^ NPs seem to be only moderately efficient multi-photon absorbing luminophores.

## Conclusions

We have investigated the correlation between the size and shape of colloidal Gd_2_O_3_ NPs and the Eu^3+^ luminescence. In the case of lanthanide-doped inorganic NPs, the size and shape effect on spectroscopic features is more ambiguous, than for QDs or metallic NPs. Surface to volume ratio, type of surface attached ligands molecules, as well as deliberately introduced defects in the crystal structure of NPs can have great influence of their optical properties. For the purpose of that work, we have obtained Gd_2_O_3_:Eu^3+^ NPs shaped as nanospheres, nanodiscs, nanotripods, and nanotriangles. Narrow size and shape distribution of obtained Gd_2_O_3_:Eu^3+^ NPs allowed us to relate the observed changes in Eu^3+^ luminescence with morphology of NPs. The initially observed narrowing and splitting of ^5^ *D*_0_ → ^7^ *F*_J_ transitions for bigger ~40 nm NPs (nanotripods and nanotriangles) could be attributed to the changing homogeneous crystal fields, when compared to the smaller ~5 nm in size Gd_2_O_3_ NPs. In such small NPs, the crystal field changes significantly from grain to grain due to the higher dispersion of lattice constants. In addition, a higher number of Eu^3+^ ions are located close to the surface of the Gd_2_O_3_ particle. It is expected that those surface states influence the energies and splitting of ^5^ *D*_0_ → ^7^ *F*_J_ transitions as well. This effect is more important for a small diameter particle (~ 5 nm) than for a bigger one (~40 nm), due to the higher probability of the Eu^3+^ ions being close to the surface of Gd_2_O_3_ grains, if we consider only random distribution of atoms in the Gd_2_O_3_:Eu^3+^ nanomatrix. Additionally, a high number of Eu^3+^ ions located near or at the surface of the investigated colloidal nanosized Gd_2_O_3_ resulted in emission spectra with shapes similar to those of the monoclinic form of Gd_2_O_3_ NPs. Such ions demonstrate a lower coordinating number, and behave as if they were embedded in lower symmetry sites, causing noticeable changes in the shape of emission spectra. Based on emission spectra and kinetics, we have estimated the optimum Eu^3+^ ions doping level concentration in the host matrix. The higher optimum (3 and 4 %) europium (III) concentrations were found for smaller nanosphere and nanodiscs shaped Gd_2_O_3_:Eu^3+^ NPs in comparison with 0.5 and 1 % for nanotripods and nanotriangles. Those differences were also related to the size of the studied NPs. The spectroscopic properties of the synthesized Gd_2_O_3_:Eu^3+^ NPs were also evaluated in the frame of Judd–Ofelt theory. The intensity parameter *Ω*_2_ showed lower hypersensitive behavior of nanospheres and nanodiscs, and also indirectly proved the incorporation of Li^+^ ions inside the Gd_2_O_3_ crystal matrix. The hypersensitive character of *Ω*_2_ parameter resulted also from the colloidal character of synthesized Gd_2_O_3_:Eu^3+^ NPs, and interaction between Eu^3+^ ions situated close to the NP surface and capping ligands molecules. Additionally, both radiative rates and quantum efficiencies were higher for the nanotripods and the nanotriangles. Finally, we have investigated the NLO properties of differently shaped Gd_2_O_3_:Eu^3+^ NPs. The smallest, nanosphere shaped, Gd_2_O_3_:Eu^3+^ NPs did not show any measurable NLO absorption in the whole spectral range. However, we have observed NLO absorption in the bigger Gd_2_O_3_:Eu^3+^ NPs in the spectral range between 600 nm and 750 nm. The strength of the NLO absorption scaled with the Gd_2_O_3_:Eu^3+^ NPs size, and was the highest for the nanotripod-shaped particles. By calculating the *σ*_2,eff_/M merit factor, and comparison with other lanthanide-doped NPs, quantum dots, and metallic NPs, we have concluded that the investigated Gd_2_O_3_:Eu^3+^ NPs are from ten to a hundred times less efficient than efficient two-photon absorbing chromophores.
